# Drug Repurposing of FDA Compounds against α-Glucosidase for the Treatment of Type 2 Diabetes: Insights from Molecular Docking and Molecular Dynamics Simulations

**DOI:** 10.3390/ph16040555

**Published:** 2023-04-06

**Authors:** Rebwar Saeed M. Rashid, Selin Temurlu, Arwa Abourajab, Pelin Karsili, Meltem Dinleyici, Basma Al-Khateeb, Huriye Icil

**Affiliations:** 1 Department of Chemistry, Faculty of Arts and Science, Eastern Mediterranean University, Famagusta 99628, Northern Cyprus, Mersin 10, Turkey; 2 Department of Chemistry, Faculty of Education, University of Sulaimani, Sulaymaniyah 46001, Iraq

**Keywords:** drug repurposing, diabetic mellitus, α-glucosidase, molecular docking

## Abstract

Type 2 diabetes mellitus is a chronic health problem that can be controlled by slowing one’s carbohydrate metabolism by inhibiting α-glucosidase, an enzyme responsible for carbohydrate degradation. Currently, drugs for type 2 diabetes have limitations in terms of safety, efficiency, and potency, while cases are rapidly increasing. For this reason, the study planned and moved towards drug repurposing by utilizing food and drug administration (FDA)-approved drugs against α-glucosidase, and investigated the molecular mechanisms. The target protein was refined and optimized by introducing missing residues, and minimized to remove clashes to find the potential inhibitor against α-glucosidase. The most active compounds were selected after the docking study to generate a pharmacophore query for the virtual screening of FDA-approved drug molecules based on shape similarity. The analysis was performed using *Autodock Vina* (ADV)—based on binding affinities (−8.8 kcal/mol and −8.6 kcal/mol) and root-mean-square-deviation (RMSD) values (0.4 Å and 0.6 Å). Two of the most potent lead compounds were selected for a molecular dynamics (MD) simulation to determine the stability and specific interactions between receptor and ligand. The docking score, RMSD values, pharmacophore studies, and MD simulations revealed that two compounds, namely Trabectedin (ZINC000150338708) and Demeclocycline (ZINC000100036924), are potential inhibitors for α-glucosidase compared to standard inhibitors. These predictions showed that the FDA-approved molecules Trabectedin and Demeclocycline are potential suitable candidates for repurposing against type 2 diabetes. The in vitro studies showed that trabectedin was significantly effective with an IC_50_ of 1.263 ± 0.7 μM. Further investigation in the laboratory is needed to justify the safety of the drug to be used in vivo.

## 1. Introduction

Type 2 diabetes is a chronic metabolic disorder widely spread in developed countries, and has become a widespread and rising issue in these communities [1]. It is related to hyperglycemia and affects roughly 537 million people worldwide, accounting for 7.2% of the global population. Of all diabetic patients, more than 95% suffer from type 2 diabetes. Diabetes is the ninth leading cause of mortality, accounting for approximately 6.7 million deaths [2,3]. Type 2 diabetes is equally prevalent in all genders. Approximately 240 million people with diabetes are undiagnosed, and the global healthcare cost for diabetics was USD 966 billion in 2021 for adults aged 20−79 [4]. Poor diet, lack of physical activity, smoking, stress, ageing and oversleeping also increase the chances of diabetes [5]. Diabetic patients are more prone to health conditions, including high blood pressure, high cholesterol, cancer, heart failure, and stroke.

The α-glucosidase enzyme found on the surface of the small intestine aids in carbohydrate digestion, producing glucose for intestinal absorption, which in turn leads to increased blood glucose levels. The inhibition of the α-glucosidase enzyme in patients with type 2 diabetes may reduce hyperglycemia; thus, the carbohydrate metabolism slows down. This study uses α-glucosidase as the target protein to predict drug candidates against it for inhibition. α-glucosidase inhibitors should be safe and capable of decelerating glucose absorption, and consequently decreasing hyperglycemia [6].

Orally administered anti-diabetic drugs act through different mechanisms in controlling blood glucose levels. A combination of drugs has also been broadly used to increase treatment efficacy [7]. There are already some drug candidates against α-glucosidase used to treat type 2 diabetes. Hitherto, these drugs have absorptive problems, inhibit other non-intestinal proteins [8,9] and long-term side effects including cardiovascular diseases, liver disorders, hypoglycemia, lactate acidosis, and gastrointestinal complaints such as abdominal distension, diarrhea, and drug resistance [7,10]. Acarbose, Miglitol, and Voglibose maintain the glucose levels to slow down the digestion of carbohydrates, delay glucose absorption in the blood, and are used as first-line drugs for treating type 2 diabetes [11]. To deal with type 2 diabetes, these drugs bind to the carbohydrate-binding regions at α-glucosidases and delay oligosaccharides’ cleavage to monosaccharides, and additionally help in reducing cardiovascular disorders and obesity [12,13]. Moreover, numerous drugs have been discovered in silico, with active biomolecules interacting with the target sites of complex molecular structures in the human body to obtain specificity and efficacy [14].

New effective α-glucosidase inhibitors are highly required due to the side effects, safety, and absorptivity issues associated with currently available inhibitors. Notably, drug repurposing is gaining popularity as a quick and effective method of identifying new services of approved drugs unrelated to their original medical intent, and is successfully moving towards the second phase of clinical trials. In this study, we moved toward drug repurposing with computational methods of drug design, using FDA-approved drugs. We aimed to use this widespread approach and develop drug candidates against α-glucosidase for type 2 diabetes within a short period and at a reduced cost. As such, in early preclinical studies, lower failure rates will demonstrate the safety profiles of the rediscovered drugs.

## 2. Results and Discussion

### 2.1. Target Protein Domains and Physicochemical Properties

**Figure 1:**
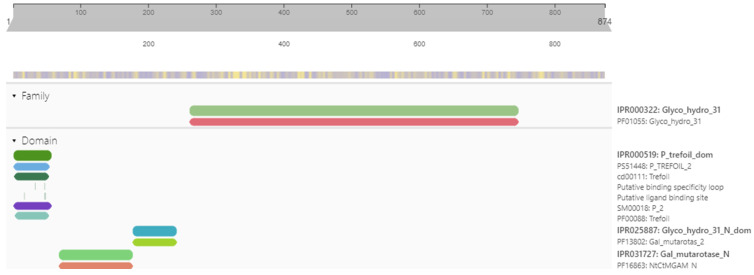
Domain architecture of α-glucosidase with three main domains and a glycoside hydrolase family.

**Figure 2:**
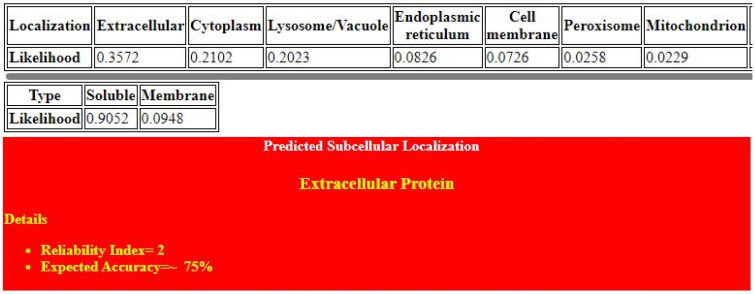
Subcellular localization of target protein via DeepLoc-1.0 and ESLpred web server.

The structure of α-glucosidase consists of 874 amino acids with a molecular weight of 101.28 KDa, a theoretical PI of 5.29, an aliphatic index of 82.89, and a −0.137 grand average of hydropathy value (GRAVY). Figure 1 shows the domain architecture of α-glucosidase, which has three main domains and belongs to the glycoside hydrolase family. The first one is the cysteine-rich domain, which is approximately forty-five amino-acid residues long and is known as the ‘P’, ‘trefoil’, or ‘TFF’ domain, composed of three loop-like regions. The beta-barrel-like structure of the gal mutarotase N domain contributes to the construction of the receptor-binding site by donating a loop that comes into close contact with two regions of the catalytic domain, thus forming the site. Gal_mutarotase_2 is found N-terminal to the glycosyl-hydrolase domain and appears similar to the galactose mutarotase superfamily. Glyco_hydro_31 hydrolyses the glycosidic bond between two or more carbohydrates or between a carbohydrate and a non-carbohydrate moiety. According to DeepLoc-1.0 and the ESLpred web server, α-glucosidase is an extracellular lysosomal protein. The expected accuracy of the ESLpred web server is approximately 75%, and the likelihood of DeepLoc-1.0 is 0.3572 (Figure 2).

### 2.2. Protein Structure Refinement, Optimization, and Minimization

**Figure 3:**
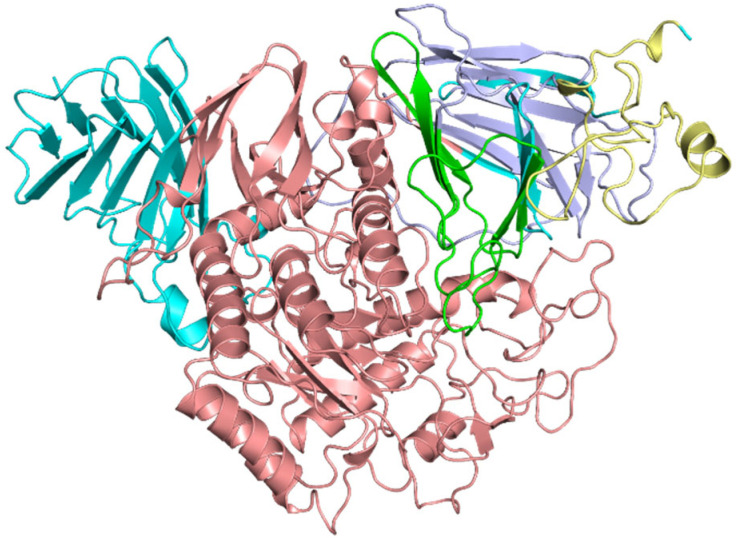
Three-dimensional structure of the target protein Alpha Glucosidase enzyme. P Terfoil domain (TFF) (2–56) is represented in pale-yellow color, Gal_mutarotase_N (69–176) in light blue, Gal_mutarotase_2 (178–241) in green, Glyco_hydro_31 (262–746) in salmon, and Glyco_hydro_b (627–874) in cyan color.

The missing residues in the structure of α-glucosidase were filled using MODELLER. The best model was selected and then minimized by two servers, Chiron and YASARA. We chose the minimized structure from Chiron instead of YASARA due to its lower energy. The total clashes and clash ratio before energy minimization were 771 and 0.0433402. After energy minimization, these values decreased to 436 and 0.0177079, respectively. Figure 3 shows the protein structure with highlighted domains in different colors. The RMSD values between the original structure and the structure after filling in the missing residues is 0.293, indicating a high degree of similarity between the two structures.

**Figure 4:**
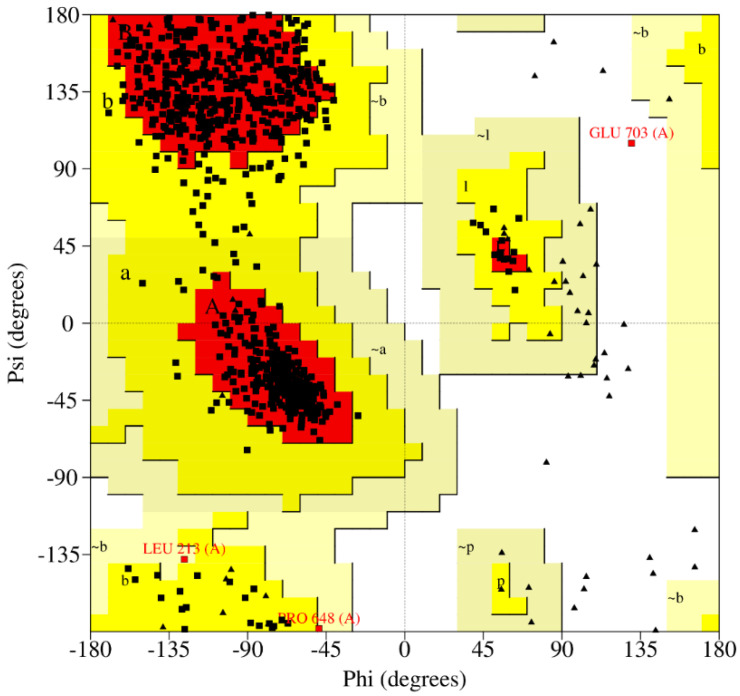
Ramachandran plot of Alpha Glucosidase, which represents most favored and favored regions and additional allowed regions.

The PROCHECK analyses the stereochemistry of the target protein and generates a Ramachandran plot (Figure 4). According to the plot, 88.4% of residues are in the most favored regions, 11% in additional allowed regions, and 0.1% in generously allowed and disallowed regions.

### 2.3. Reported Inhibitors Docking and Pharmacophore Query

**Figure 5:**
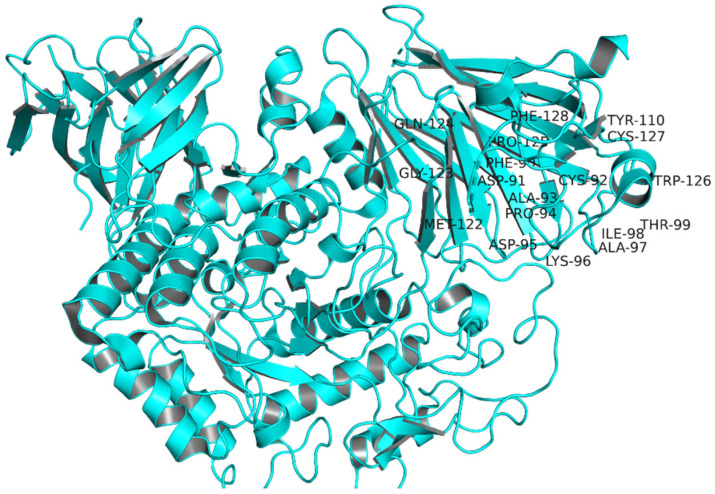
Active site residues of target protein identified via literature and CastP.

The target protein’s binding/active site residues are LYS96, ASP95, ALA97, PHE90, ILE98, THR99, CYS92, ALA93, ASP91, PRO94, MET122, PRO125, GLN124, GLY123, PHE128, TYR110, and CYS127, as labelled in Figure 5 [15].

**Figure 6:**
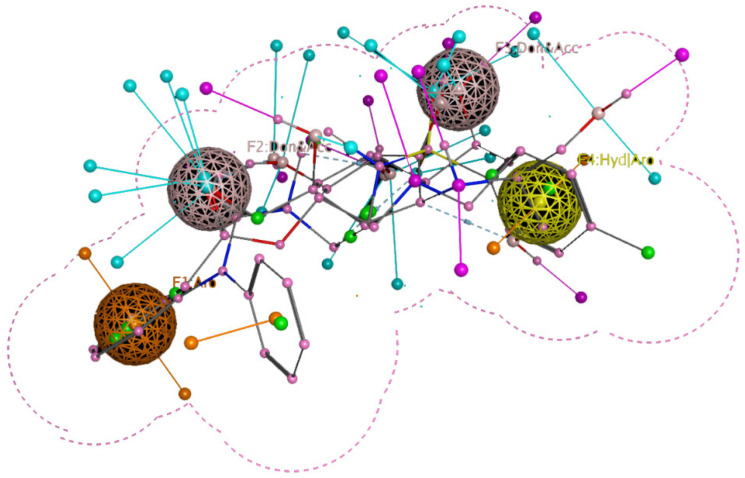
Pharmacophore query for screening purposes. Color balls represent HBA, HBD, hydrophobic, and aromatic groups.

Site-specific docking was performed on 20 reported inhibitors [16,17,18,19,20] (acarbose, celgosivir, metformin, migalastat, 4-(4-methylbenzenesulfonyl)-N,N-diphenylpiperazine-1-carboxamide, 1-Deoxynojirimycin, (6S,7R,8R)-Octahydro-indolizine-1,6,7,8-tetraol, miglitol, NAG, Voglibose, metformin, BGC, GLC, NOJ, (2R)_2alpha_Ethylpyrrolidine_3beta_4alpha_diol, SC2, GOL, PGE, PEG, EDO, and MIG) using PyRx. The most active compounds such as Celgosivir (60,734), 4-(4-methyl benzenesulfonyl)-N,N-diphenylpiperazine-1-carboxamide (1,322,817), and Voglibose (444,020) were analyzed and utilized for the pharmacophore query generation (Table 1). Flexible alignment was performed on the selected ligands to generate conformers, which helped identify the common features used to generate pharmacophore queries. Four essential features were chosen for pharmacophore query generation, such as hydrogen bond acceptor (HBA), hydrogen bond donor (HBD), hydrophobic, and aromatic groups, highlighted with different colors (Figure 6). This query was used for virtual screening to find similar compounds. The selected inhibitors have the capability to inhibit α-glucosidase activity, leading to decreased glucose release that slows down carbohydrate digestion and the absorption of glucose in the small intestine. This is a contemporary therapeutic approach for stabilizing blood glucose levels in diabetic patients, especially in type 2 diabetes.

### 2.4. Database Preparation and Virtual Screening

For virtual screening, the prepared database of FDA compounds was used. Compounds with similar pharmacophoric features were retrieved from the database using MOE for virtual screening. After the virtual screening, we obtained 11 FDA compounds that showed structural similarity to the pharmacophore query with a good pharmacophore fit score (Table 2). These compounds were then employed for molecular docking to find the best docking pose with the target protein.

### 2.5. Molecular Docking and Lead Identification

**Figure 7:**
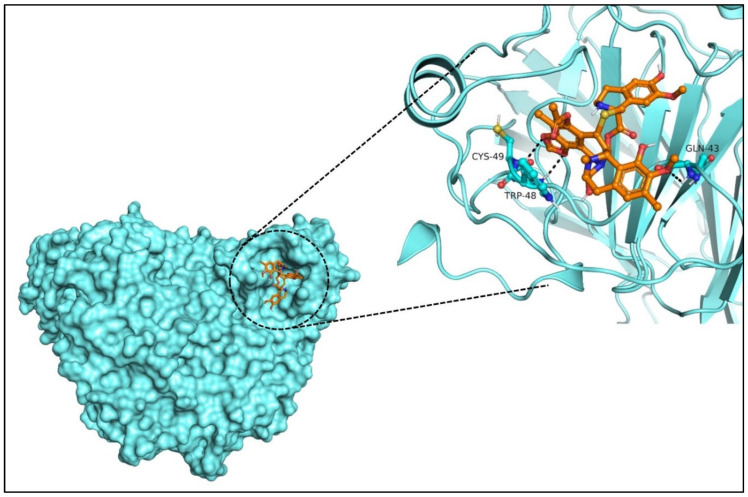
Best docked pose of most potent hit Trabectedin with Alpha Glucosidase in surface form (left image) and ligand–receptor interactions in cartoon form (right image).

**Figure 8:**
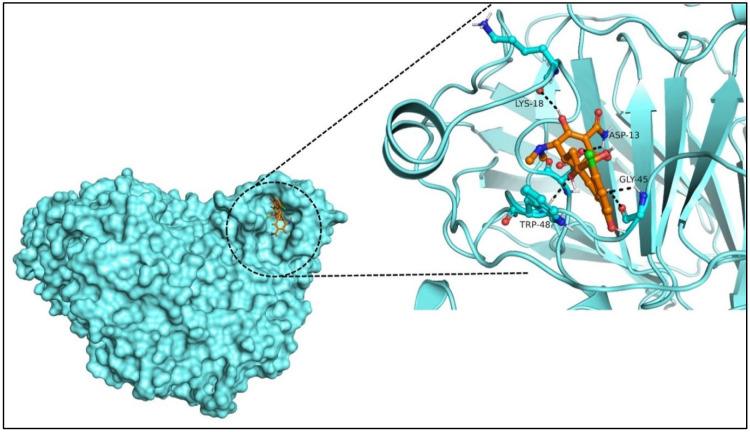
Second best-docked pose of Demeclocycline with Alpha Glucosidase in surface form (left image) and ligand–receptor interactions in cartoon form (right image).

Site-specific docking was performed for the selected 11 compounds after screening. Docking was carried out using Autodock Vina to narrow down the hits. The top 2 compounds, Trabectedin and Demeclocycline, were selected as the lead compounds because they showed good binding affinity, formed hydrogen bonds, and bound in the receptor’s active site. The results show that the lead compounds Trabectedin (Figure 7) and Demeclocycline (Figure 8) had stable binding interactions with amino acids of α-glucosides, mostly through hydrogen bonds.

### 2.6. Docking Validation via ROC Curve

Two lead compounds were superimposed with a reference compound and analyzed before the simulation for validation purposes. The reference compound (α-D-glucopyranose and β-D-glucopyranose) was obtained from the experimental structure. The ligand binds to the (Ileu98, trp126, cys127, asp91, ala93, gly123, gln124) residues with conventional hydrogen The fact that the reference and lead compounds bind at the same position indicates that the lead compound is making interactions with residues that are involved in the interaction with the reference compound. Additionally, the obtained RMSD was 0.293 Å, leading to the validation of docking results.

**Figure 9:**
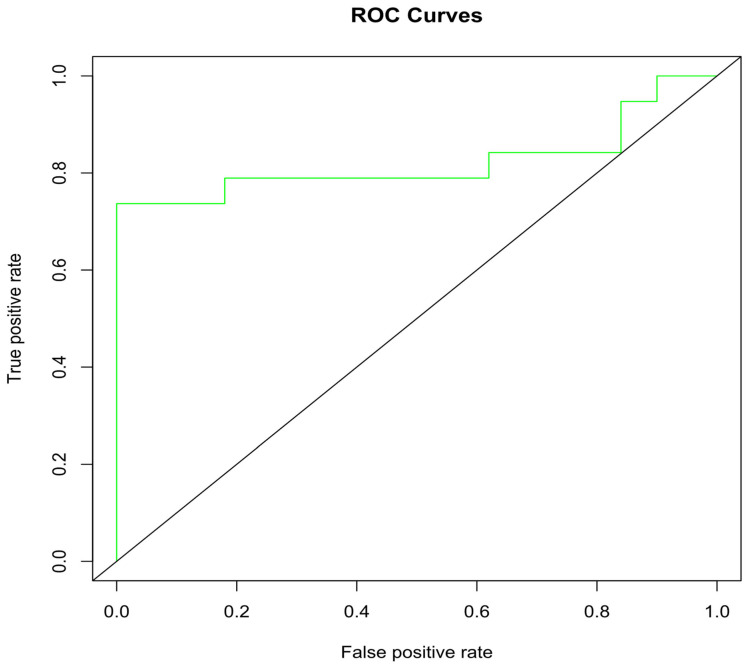
The ROC curve for the validation of docking and the area under the ROC curve calculation quantifies the overall docking results. The green line represents AUC (0.822).

The ROC (receiver operating characteristics) evaluation system was also used to check the docking validation by distinguishing active and inactive ligands (Figure 9). A ROC curve close to 1 indicates the model’s ability to make a differentiation between the active and inactive compounds, while a curve close to zero shows the opposite. Area-under-the-curve (AUC) values that lie between 0.8 and 0.9 are considered good, values between 0.7 and 0.8 are acceptable, while values 0.6 and 0.5 are considered poor. The ROC generated reliable results with an AUC value of 0.822 and a ROC curve close to 1. Therefore, these compounds were used for further analysis.

**Figure 10:**
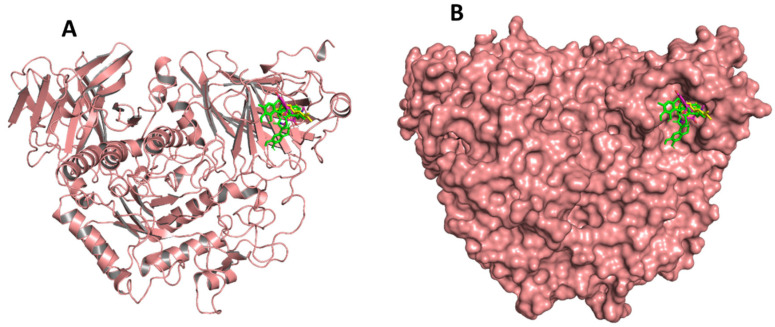
The superimposition of the lead compounds and reported active compounds in the cartoon (**A**) and surface form (**B**). All compounds make interactions with active site resides.

Before simulation, two lead compounds and the active compound were superimposed and analyzed. The active compound and lead compounds bind in the active site and interact with different residues, as shown in Figure 10 in cartoon and surface form.

### 2.7. Molecular Dynamics Simulations

MD simulations were performed for both complexes (100 ns) using the Desmond Simulation Package. MD trajectories were used to determine root mean square deviation (RMSD) values, root mean square fluctuation (RMSF) values, and protein–ligand contacts. The MD simulations for two complexes were run with the same parameters for comparison. Notably, the results for each complex were reproducible.

**Figure 11:**
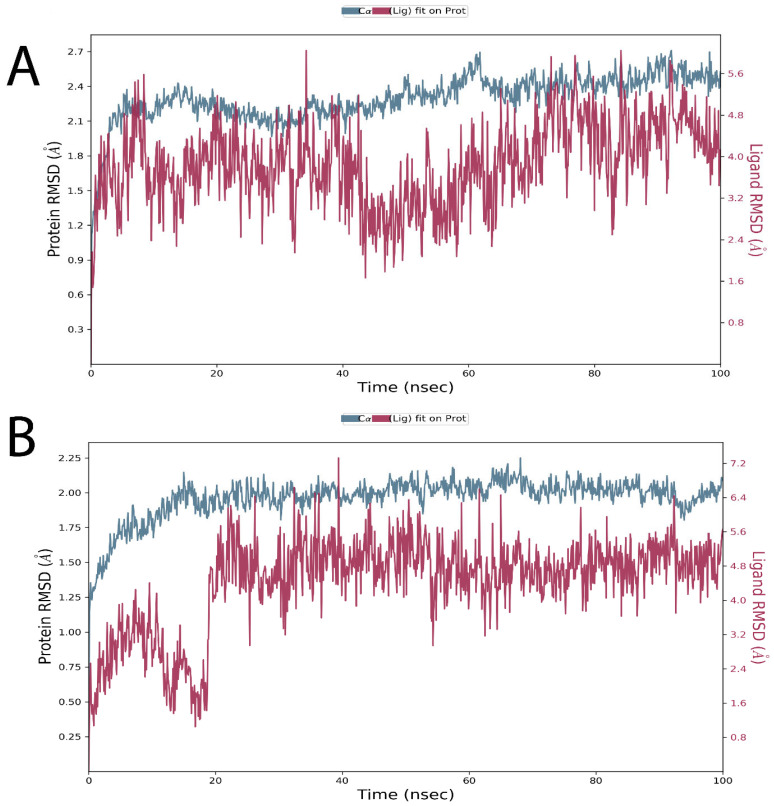
(**A**) RMSD of the Cα atoms of α-glucosidase and the ligand Trabectedin with time (**B**) RMSD of the Cα atoms of α-glucosidase and the ligand Demeclocycline overtime. The left y axis represents the RMSD variations of the Cα atoms of the target protein, and the right y axis represents the ligand’s RMSD variations (red color) over time.

Figure 11A,B show the RMSD values over time for Cα atoms of Trabectedin and Demeclocycline binding with the target protein over time. RMSD measures the stability of protein and ligand backbone atoms (Cα) and depends on the interaction and energy between the protein and ligand when they are in complex form. The RMSD plots show the protein RMSD at the left Y axis and ligand RMSD at the right Y axis. Desmond takes the first frame as a reference frame, then aligns all protein frames on the reference frame’s backbone and calculates the RMSD. The protein RMSD provides structural conformation throughout the simulation, while ligand RMSD provides ligand stability in the protein’s binding pocket. Figure 10A indicates that the protein is stable at 5 ns with an average of 2.1 Å, which persists up to 60 ns, and at 62 ns, there is a fluctuation. However, it is not a significant change, and the protein is stable throughout the time within 2.0–2.4 Å. At 10 ns, the ligand is stable. Then, a more extended peak arises at 35 ns due to any flip or rotatable bond. Again, it becomes stable for the rest of the simulation within 2.4  ±  5.0 Å, and this RMSD is significant, which might be due to the interaction between the ligand and the other residues in the receptor and lead to conformational change as the ligand is flexible, or it might experience a strong fluctuation force. Nevertheless, the ligand remains in the binding site and stays stable for the rest of the simulation. No significant change in ligand RMSD indicates that the ligand did not leave the protein’s binding pocket. The range of ligand fluctuations is higher than the protein due to numerous rotatable bonds in the ligand, but these are acceptable. In Figure 10B, at 15 ns, the protein is stable for the rest of the simulation within 2.0 to 2.4 Å, while ligand RMSD is stable at 20 ns throughout the simulation within 1.6 to 6.4 Å.

**Figure 12:**
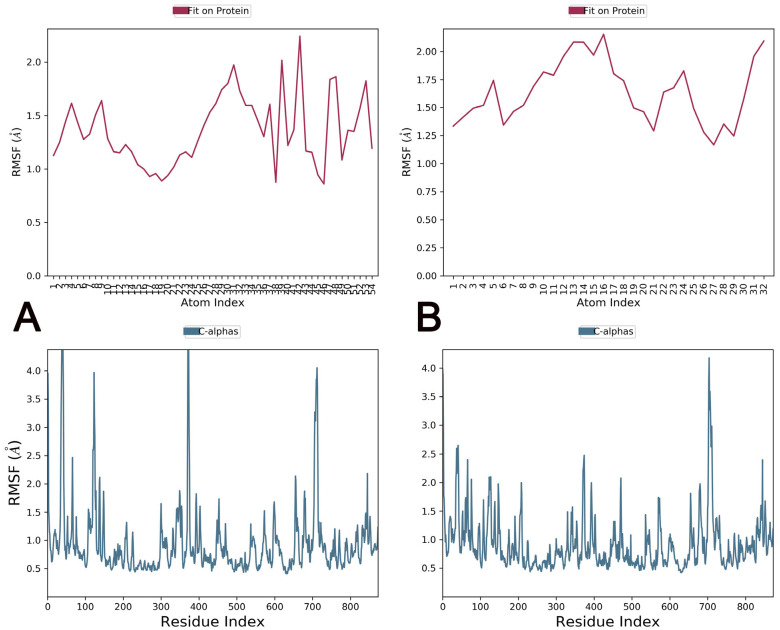
Upper panel: atom-wise RMSF of the Trabectedin ligand (**A**) and Demeclocycline ligand (**B**) with respect to the target protein (α-glucosidase); Lower panel: residue-wise RMSF of α-glucosidase during simulation.

Figure 12A,B show atom-wise (ligand) and residue-wise (receptor) root mean square fluctuation (RMSF) variations of Trabectedin and Demeclocycline. RMSF calculates individual residue flexibility during simulation. Due to more fluctuation, the residues at N- and C-terminals are free, and four peaks in the Figure 12A lower panel and one in 11 B show higher variations in the RMSF value, indicating coil or loop regions. In contrast, lesser fluctuation peaks show α-helices or β-sheets, and the areas with very minute fluctuations are binding site residues that are primarily conserved. Except for the higher loops, most residues of α-glucosidase maintain an RMSF value <2.5 Å. The RMSF of both ligand atoms with respect to the α glucosidase is maintained at <2.0 Å for most atoms. RMSF local fluctuations based on the composition of secondary structures such as helixes (15.17%) and β-strands (25.23%) of 40.41% and 59.59% are loops and coils that exhibit higher fluctuations.

**Figure 13:**
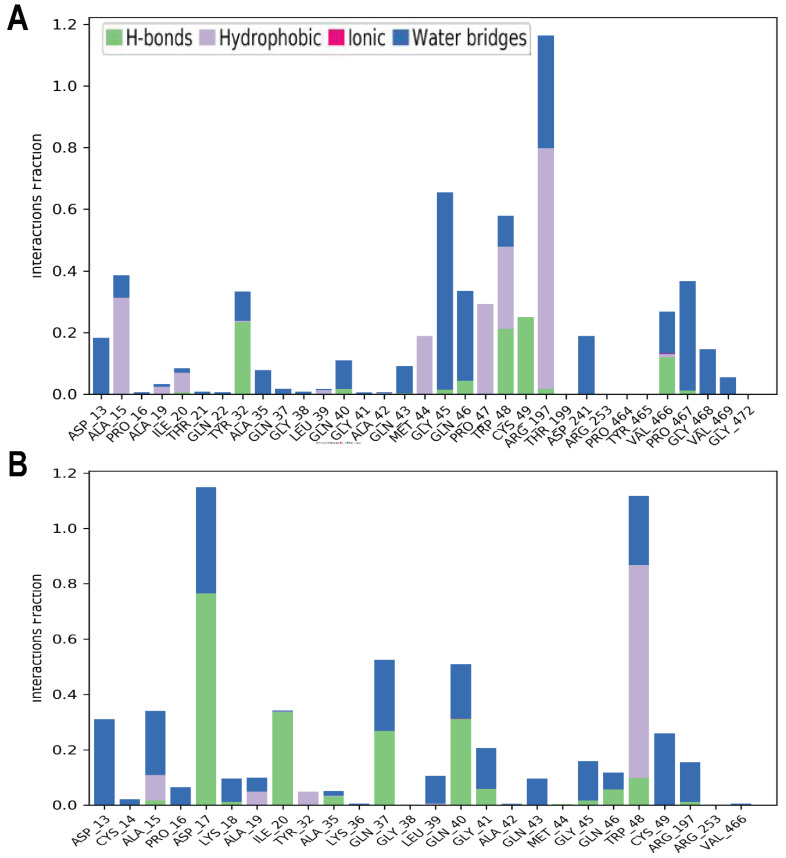
(**A**) Summary of all contacts of α-glucosidase and Trabectedin (**B**) all contacts of α-glucosidase and Demeclocycline during MD simulations.

Figure 13A,B show interactions during MD simulations between the residues of α-glucosidase and the atoms of Trabectedin and Demeclocycline ligands. Trabectedin mainly interacts through water bridges, hydrogen bonds, and hydrophobic interactions (Figure 12A). The hydrophobic interactions are primarily shown by nonpolar and positively charged compounds such as Ala15, Ile20, Met44, Pro47, Trp48, and Arg197. The most representative water bridges are formed by Gly45 and Gln46, Arg197, and Pro467 residues throughout the simulation period. Demeclocycline shows interactions by hydrogen bonds, water bridges, and then hydrophobic interactions (Figure 13B). The hydrogen bonds are mainly generated by polar and negatively charged amino acids such as Asp17, Ile20, Gln37, and Gln40 residues. Water bridges are formed by polar and negatively charged amino acids such as Asp13, Asp15, Gln37, and Gln40 residues throughout the simulation period, and Trp48 shows hydrophobic interactions. Notably, these interactions are responsible for ligand stabilization within α-glucosidase.

**Figure 14:**
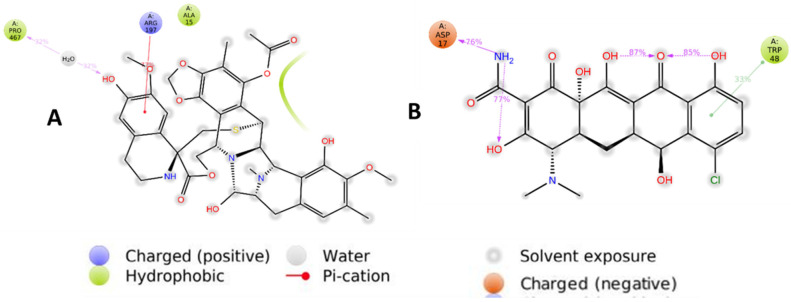
An interaction of less than 30.0% observed during the total simulation period between lead compounds Trabectedin (**A**), Demeclocycline (**B**), and the receptor.

The most important hydrogen bonds generated by Ser65, Ser66, Ile107, Arg154, and Ser396 residues remained present for more than 50% of the simulation time. PRO467 and ASP17 show hydrogen bond interactions, while ARG197 and TRP48 make Pi-cation interactions (Figure 14). These interactions are responsible for binding and stabilizing ligands within the active site of α-glucosidase with good binding affinities.

### 2.8. Binding Free Energy (MMGBSA) Calculation

**Figure 15:**
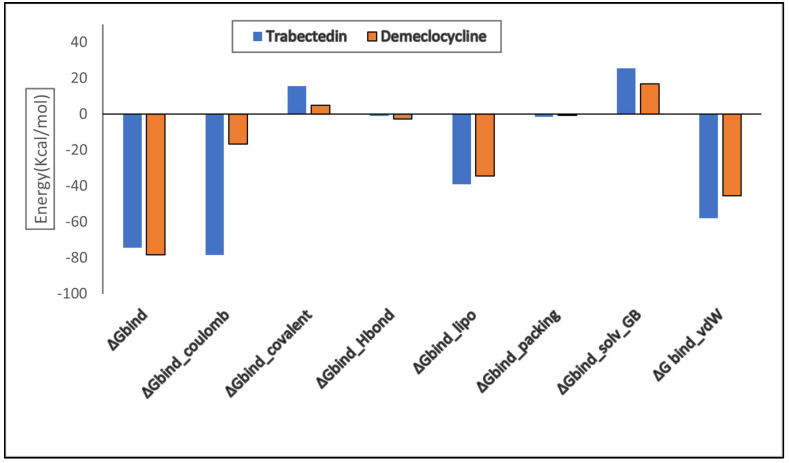
Histogram of calculated binding free energy values of the two best compounds in contact with receptor via the MM-GBSA method.

The MMGBSA technique is computationally efficient, cost-effective, takes less time, and is used to compute the binding free energy of small molecules fit into macromolecules. The estimated binding energies for both compounds are shown in Table 3 and the graph (Figure 15). Computed energy profiles include Coulombic, covalent, and lipophilic contributions. Van der Waals provides molecular indications that may benefit drug design and development. Deviations in binding free energy (ΔG_bind_) are (−74.36 Kcal/mol) for Trabectedin and (−78.31 Kcal/mol) for Demeclocycline, representing a −3.95 variation. Lower binding free energy denotes greater stability and the favorable binding of the ligand to the protein. According to binding free energy values, Van der Waals contributes more than the others. Prime MM-GBSA accurately calculates the binding energies and demonstrates a robust interaction between the ligand and macromolecule. 

### 2.9. Alpha-Glucosidase Assay

This study aimed to investigate the inhibitory effects of two leading compounds (Trabectedin and Demeclocycline) on α-glucosidase using acarbose as a positive control. The IC_50_ value for acarbose was determined to be 1.258 mM, while Trabectedin exhibited a greater inhibition activity and a notable increase in inhibition activity with increasing concentration. The IC_50_ value for Trabectedin was found to be 1.263 ± 0.7 μM, whereas Demeclocycline did not show a significant effect. It has been reported that Trabectedin interferes with DNA and binds to the minor groove of DNA, inhibiting the cell proliferation [21]. Nevertheless, the relationship between anticancer treatments and diabetes is complex. Some treatments can worsen glucose control, while others may have potential antidiabetic effects. Therefore, the close monitoring and individualized management of diabetic patients undergoing anticancer therapy is essential to optimize the treatment outcomes [22]. Hence, further investigation is recommended to assess the potential side effects of Trabectedin as it is an anticancer agent and warrants in vivo studies.

## 3. Material and Methods

### 3.1. Target Protein and Its Properties

α-Glucosidase is used as a target protein for type 2 diabetes, following a literature review. Its accession number is NP_000143.2 and its UniProt ID is P10253. These IDs are used to retrieve the sequence, function, and other information about the target protein. The physicochemical properties of the target protein, such as its molecular weight, aliphatic index, number of atoms and atomic composition, theoretical PI (isoelectric point), grand average of hydropathicity (GRAVY), and negatively and positively charged atoms, are retrieved from the Expasy ProtParam (https://web.expasy.org/protparam/, accessed on 10 March 2022), InterPro (ebi.ac.uk/interpro/, accessed on 10 March 2022), and NCBI’s CD-search (https://www.ncbi.nlm.nih.gov/Structure/cdd/wrpsb.cgi, accessed on 10 March 2022) to find domains, family, superfamily, and essential sites. The subcellular localization of the target protein is found using the DeepLoc-1.0 and ESLpred web servers. Both tools use protein characteristics such as amino acid composition, physicochemical properties, and dipeptide composition. Support vector machine (SVM)-based algorithms use these characteristics to predict the subcellular localization of eukaryotic proteins [15].

### 3.2. Protein Structure Refinement, Optimization, and Minimization

The protein structure of α-glucosidase is already available in the Protein Data Bank (PDB) with one unique protein chain, 2-angstrom resolution, and its R-free value is 0.185. The X-ray diffraction method is used for α-glucosidase, and the PDB ID is 5KZW [23]. Twenty-four missing residues are filled by MODELLER and then minimized by two different servers, Chiron and YASARA (http://www.yasara.org/minimizationserver.htm, accessed on 11 March 2022). More energetically favorable structures are obtained upon energy minimization. Chiron is an automated web server that identifies and resolves the steric clashes of proteins by defining clashes based on the Van der Waals repulsive energy of the clashing atoms [24]. The protein structure is validated via PROCHECK by creating Ramachandran Plot.

### 3.3. Retrieval of Reported Inhibitors and Docking

The already-reported inhibitors/active compounds were retrieved from the PubChem database and the literature, as they will help generate a pharmacophore query on the basis of structural similarities. For site-specific docking, active, binding, and conserved sites were decided on via literature search and CASTp web server (http://sts.bioe.uic.edu/castp/index.html?1ycs, accessed on 12 March 2022). CASTp provides the detailed topographic characteristics of proteins, such as surface pockets and internal cavities, which are essential for protein function [25]. The best binding site was picked with the highest probability. For docking purposes, 20 active molecule’s structures retrieved by PubChem were converted into PDB and then into pdbqt file format using Autodock. The macromolecule is prepared by removing water molecules and adding polar hydrogens and charges. The site-specific docking was performed via PyRx and analyzed via PyMOL.

### 3.4. Pharmacophore Query, Database Preparation, and Virtual Screening

The docking of reported inhibitors was analyzed based on docking scores and RMSD values. All the compounds were aligned by the Molecular Operating Environment (MOE) to find structural similarities and merge them to generate pharmacophore. The database of 1600 FDA-approved drugs was prepared and used for screening purposes, as these compounds were selected for pharmacokinetic and safety purposes. FDA-approved drugs were retrieved from the ZINC database (https://zinc15.docking.org/, accessed on 15 March 2022). The MOE (pharmacophore search protocol) was used to perform virtual screening based on the generated pharmacophore query.

### 3.5. Molecular Docking, Validation, and Lead Identification

Molecular docking is a method in drug design to achieve the best conformation of the ligand–protein complex. It was executed via Autodock Vina, an open source docking tool. Small molecules were downloaded in SDF file format and converted into PDB via Open Babel software [26]. Before docking, both ligand and protein were preprocessed and further prepared via Autodock; water was removed from macromolecule, polar hydrogens were added, charges were assigned and torsions were selected in case of ligand. The grid box was adjusted around active site for site-specific docking. The dimensions of X, Y and Z coordinates were 63.750, 87.139 and 71.694, respectively, while the box size was 54 × 56 × 50 for all three dimensions. Ligand bonds were made rotatable and then the ligand and protein were converted into PDBQT format. PyMOL (http://www.pymol.org/pymol, accessed on 25 March 2022) was used to visualize ligand–protein interactions. For docking validation, the co-crystalized ligand (NAG) inside the PDB ID 5KZW receptor was extracted and searched on the ChEMBL (https://www.ebi.ac.uk/chembl/, accessed on 10 March 2022) site, and 20 compounds were selected on the basis of an IC50 value. The dataset of 50 decoys was obtained via DUD•E (http://dude.docking.org/, accessed on 5 June 2022). All the compounds were docked by incorporating same conditions used for the investigated ligands of this study, and on the basis of docking results, active and decoys were used to draw the ROC curve. After a detailed analysis of ligand–receptor interactions, their binding affinity, RMSD, and Ph4 (pharmacophore) Score, the most active compounds were selected and then carried out for MD simulations.

### 3.6. MD Simulations and Binding-Free Energy (MMGBSA) Calculation

The top two compounds were selected as lead compounds and further validated by the molecular dynamic simulation of 100 ns using Desmond, the Package of Schrödinger [27]. Molecular docking studies predicted the binding of the ligand under static conditions, while simulations predicted ligand binding under physiological conditions. The protein–ligand complexes were solvated with SPC explicit water molecules and placed in the center of an orthorhombic box of an appropriate size under the periodic boundary conditions. Protein–ligand complexes were preprocessed, including optimization, equilibration, and minimization, using the Protein Preparation Wizard of Maestro default parameters. The system prepared using the system builder tool and OPLS 2005 force field was used in the simulation to describe molecular geometry [28]. During simulation, the models were neutralized to obtain the correct electrostatic values by adding counter ions such as Na^+^ or Cl^−^ (0.15 M NaCl) to simulate the physiological conditions. Before starting the MD simulation, bad connections were eliminated, and the system’s deformed geometries and steric clashes were repaired during the energy minimization step to avoid any significant steric overlap. In the minimization procedure, the entire system was allowed to relax for 2500 steps by the steepest descent approach. A temperature of 300 K with 1 atm pressure was maintained for 100 ns simulation. Before the simulation, the models were equilibrated. The equilibrated structure was used as a starting point, and a 100 ns long MD simulation was run. This phase is considered the production run. During the simulation, every 1000 ps of the actual frame was stored. The trajectories were saved for examination, and the stability of the complex was assessed by analyzing the results over time. MMGBSA analysis was performed for the two best compounds, Trabectedin and Demeclocycline, via Prime, a package of Schrödinger [29]. This approach was used to determine the binding strength and stability of the protein and ligand, to predict the binding modes, and to differentiate between the binders and non-binders. The binding strength is determined by the binding free energy (ΔGbind) for proteins and ligands via Equation (1).

ΔGbind = Gcomplex − Gprotein – Gligand(1)

ΔGbind = ΔEvdw + ΔEele + ΔEGB + ΔESA − TΔS(2)

According to Equation (2), the total binding free energy of protein and ligand is ΔGbind. While ΔEvdw and ΔEele are the van der Waals and Coulombic energy, respectively. ΔEGB, ΔESA, and TΔS represent the generalized born electrostatic energy, nonpolar energy, and conformational entropy, respectively.

### 3.7. Chemicals and Reagents

The α-glucosidas, along with dimethyl sulfoxide (DMSO), was acquired from Shanghai Trading Co., Ltd. (Shanghai, China). P-nitrophenyl- α-D-glucose (p-NPG) and Phosphate buffer were obtained from Shanghai Huacheng Industrial Development Co., (Xiamen, China). Enzyme inhibition studies (IC_50_) were conducted using a 96-well microplate spectrometer (Molecular Devices Spectra Max i3X)

### 3.8. α-glucosidase Inhibition Assay

The assay for the inhibition activity of α-glucosidase was conducted according to the method described by Chen et al. [30]. In brief, 160 µL of phosphate buffer (pH 6.8) and 10 µL of α-glucosidase were added to a 96-well plate. Then, 10 µL of each compound (Trabectedin and Demeclocycline separately) dissolved in dimethyl sulfoxide (DMSO) was added to the 96-well plate, followed by incubation at 37 °C in a microplate reader for 20 min, with oscillation for 10 s every 2 min. Meanwhile, *p*-nitrophenyl-β-glucopyranoside (pNPG) at a concentration of 10 mM was pre-incubated at 37 °C in a water bath. Finally, 20 µL of pNPG was added to the mixture to measure the change in absorbance at 405 nm with an interval of 2 min. The experiment was carried out in triplicate. As the number of products produced has a linear relationship with the absorbed light, the reaction rate of the enzyme can be obtained by measuring the change in the light absorption value per unit of time in a specific period. The percent inhibition was calculated using the following equation:

(3) $$\alpha \text{-}\mathrm{glucosidase}\;\mathrm{activity}\;(\%)=\frac{A_{sample}}{A_{control}}\times 100$$

where *A* is the absorbance growth slope, *A_control_* is the enzyme activity without the inhibitor, and *A_sample_* is the enzyme activity with the inhibitor. The data obtained were used to determine the inhibitor concentration that inhibited 50% of the enzyme activity (IC50) using the GraphPad Prism 9.1.1 software (GraphPad Inc., La Jolla, CA, USA).

## 4. Conclusions

α-glucosidase is considered an essential target for type 2 diabetes that is necessary to absorb digestive carbohydrates such as glucose and fructose and create hyperglycemic conditions. An average blood glucose level is required for the coordinated function of multiple tissues and organs. Reduced glucose synthesis and lower intestinal glucose absorption can reduce the severity of type 2 diabetes. Therefore, a library of FDA-approved compounds is used to find inhibitors against α-glucosidase, to control hyperglycemic conditions and act as a promising anti-diabetic compound with few or no side effects. Drug repurposing is a quick and low-cost method of discovering new modes of action for approved drugs for other diseases, and it was approved by both the FDA and EMA.

In this study, a total of 1600 FDA-approved drugs were screened via an in silico approach to identify the candidate compounds showing anti-diabetic effects against α-glucosidase. According to the results, Trabectedin and Demeclocycline, the two FDA-approved compounds, stably bind in the active site of α-glucosidase and show effective inhibition activities. Patients with type 2 diabetes commonly have an increased risk developing cancer and cardiovascular diseases. It is noteworthy that one of the lead compounds, Trabectedin, is a marine-derived antitumor agent and constitutes an FDA-approved treatment for soft tissue sarcoma. Moreover, with FDA approval, the second lead candidate, Demeclocycline, is used to treat Lyme disease, bronchitis, urinary tract infections, malaria, and bacterial infections.

Implementing a bioinformatic approach to investigate FDA-approved drug molecules via a drug repurposing approach for treating type 2 diabetes is a valuable research technique that can reduce the cost and shorten the research time. In this study, molecular docking indicated the strong binding interactions of FDA-approved compounds with α-glucosidase, which showed stronger interactions than the standard inhibitors of the enzyme. The docking results were validated via an ROC curve. In the ROC curve, the graphical representation of the docking performance provided insights into the AUC which is used to measure test performance. There is an 82% chances that selected compounds are active rather than inactive. The two best selected compounds were also validated via a superimposition with a reference compound. Both the selected and reference compounds bind to the same position. The binding of these compounds was further confirmed through MD simulation studies.

Trabectedin and Demeclocycline show potential for treating type 2 diabetes. In addition, the IC_50_ of trabectedin was found to be significantly lower with 1.263 μM. Moreover, only further actual in vivo experiments studies can elicit the potential of this FDA-approved drug for type 2 diabetes. Researchers have stated that anticancer drugs have a high risk of increasing diabetics as a side effect, where others have found that trabectedin is more effective in breast cancer patients with diabetics [31,32]. Based on these findings, a better structure–activity relation (SAR) can be implemented to synthesize molecules with a higher activity and a probability of lowering the side effects.

## Figures and Tables

**Table 1:** Most active compounds are used to form pharmacophore queries for screening purposes.

Small Molecules	IC_50, and_ K_d_	Structure	HBA	HBD	MW (KDa)	RB	LOG P	Lipinski Violation	Publications
Celgosivir	15.95 mM	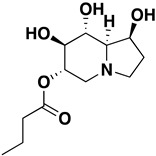	6	3	259.40	4	−0.8	NO	https://journals.sagepub.com/doi/pdf/10.1177/095632020401500304 (accessed on 12 April 2022)
4-(4-methylbenzenesulfonyl)-N,N-diphenylpiperazine-1-carboxamide	25.1189 µM	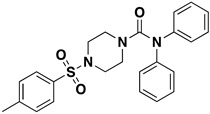	4	0	435.4	4	3.8	NO	https://pubchem.ncbi.nlm.nih.gov/compound/1322817 (accessed on 12 April 2022)
Voglibose	23.4 µM	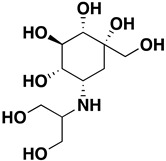	8	8	267.28	5	3.1	NO	https://pubmed.ncbi.nlm.nih.gov/15558946/ (accessed on 12 April 2022)

**Table 2:** Virtual screen output for computationally potential hits bind in the active site of α-glucosidase sorted based on binding affinity and Ph4 Score.

IDs	Drug/ Molecule Name	Structures	Binding Score	Ph4 Score
ZINC000150338708	Trabectedin	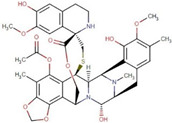	−8.8	0.490963817
ZINC000100036924	Demeclocycline	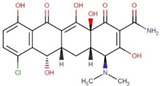	−8.6	0.64888829
ZINC000085537053	Docetaxel	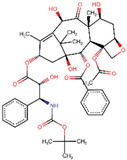	−8.6	0.780447185
ZINC000028232750	Valstar	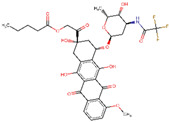	−8	0.726529598
ZINC000096006020	Paclitaxel	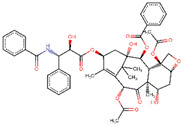	−7.9	0.727842093
ZINC000003927198	E-Cefdinir	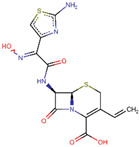	−7.8	0.501275837
ZINC000085536932	Cabazitaxel	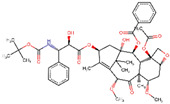	−7.7	0.719660044
ZINC000003830215	Amoxicillin	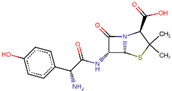	−7.5	0.82327193
ZINC000004474682	Travoprost	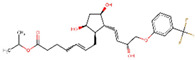	−7.4	0.78199023
ZINC000003794794	Mitoxantrone	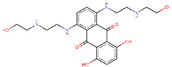	−7.2	0.807539582
ZINC000004474405	Latisse	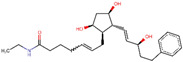	−6.7	0.685938478

**Table 3:** Binding free energy values in kcal/mol of alpha Glucosidase complexed with Trabectedin and Demeclocycline calculated via MMGBSA method.

Parameters	Trabectedin (Kcal/mol)	Demeclocycline (Kcal/mol)
ΔG_bind_	−74.36	−78.31
ΔG_bind__Coulomb	−16.15	−16.73
ΔG_bind__covalent	15.66	4.85
ΔG_bind__Hbond	−0.98	−2.78
ΔG_bind__lipo	−39.11	−34.51
ΔG_bind__packing	−1.42	−0.60
ΔG_bind__solv_GB	25.53	16.96
ΔG _bind__vdW	−57.89	−45.49

## Data Availability

Not applicable.
